# An improved border detection in dermoscopy images for density based clustering

**DOI:** 10.1186/1471-2105-12-S10-S12

**Published:** 2011-10-18

**Authors:** Sait Suer, Sinan Kockara, Mutlu Mete

**Affiliations:** 1 Computer Science, University of Central Arkansas, 201 Donaghey Ave. Conway, 72035, AR, USA; 2 Computer Science and Information System, Texas A&M University – Commerce, 75429, TX, USA

## Abstract

**Background:**

Dermoscopy is one of the major imaging modalities used in the diagnosis of melanoma and other pigmented skin lesions. In current practice, dermatologists determine lesion area by manually drawing lesion borders. Therefore, automated assessment tools for dermoscopy images have become an important research field mainly because of inter- and intra-observer variations in human interpretation. One of the most important steps in dermoscopy image analysis is automated detection of lesion borders. To our knowledge, in our 2010 study we achieved one of the highest accuracy rates in the automated lesion border detection field by using modified density based clustering algorithm. In the previous study, we proposed a novel method which removes redundant computations in well-known spatial density based clustering algorithm, DBSCAN; thus, in turn it speeds up clustering process considerably.

**Findings:**

Our previous study was heavily dependent on the pre-processing step which creates a binary image from original image. In this study, we embed a new distance measure to the existing algorithm. This provides twofold benefits. First, since new approach removes pre-processing step, it directly works on color images instead of binary ones. Thus, very important color information is not lost. Second, accuracy of delineated lesion borders is improved on 75% of 100 dermoscopy image dataset.

**Conclusion:**

Previous and improved methods are tested within the same dermoscopy dataset along with the same set of dermatologist drawn ground truth images. Results revealed that the improved method directly works on color images without any pre-processing and generates more accurate results than existing method.

## Introduction

Skin cancer is one of the most common cancer types. Three most commonly seen skin cancer types are melanoma, basal cell cancer, and squamous cell cancer which are named after the type of skin cells from which cancer arises [1]. Skin cancer is the most commonly diagnosed cancer and rarely fatal, except for melanoma [2]. Melanoma is the most rapidly increasing cancer in the world and is the sixth most common cancer in the U.S [4]. In 2010, there were estimated 68,130 new cases in the US. Unfortunately, an estimated 8,700 of these cases were fatal [3]. Although survival rate is increasing, death rate from malignant melanoma is exponentially increasing as well [4]. Early diagnosis is crucial for the treatment, because malignant melanoma is very invasive when it affects melanocyte. Melanoma develops in the epidermis. An often-used mnemonic for early signs of melanoma is "ABCDE", where A corresponds to asymmetry, B is borders (irregular), C is color, D corresponds to diameter (greater than 6 mm –0.24 inch), and E corresponds to evolving over time. Since it is found between the outer layer of the skin (the epidermis) and the next layer (the dermis), it is clearly visible by human eyes. Therefore, the diseased area can be cured by using a surgical excision operation.

Dermoscopy is one of the major imaging techniques for detecting skin lesion area. It is found that, by using dermoscopy techniques, the sensitivity of finding the lesion area increases up to 20% [5]. Dermoscopy images give dermatologists confidence in determining the lesion. Combining dermoscopy techniques and computer aided diagnosis (CAD) techniques is a very important research field. In order to prevent time loss and intra- and inter-observer variations, researchers try to utilize computerized techniques. The borders of most melanomas are often indistinct which make visual identification very difficult. Over time, the lesion may grow or the pigmentation in the lesion may darken. The previous border of the same lesion region must be compared with the current border of the same lesion side by side to evaluate it. Therefore, dermoscopy and drawing lesion borders on dermoscopy images are critical. In current practice, dermatologists visually check the dermoscopy images and draw the lesion border manually which is a tedious process. Moreover, the delineated lesion border drawn by different dermatologists may not be the same. Sometimes this difference may reach 24% [6]. This is the motivation for CAD techniques to help dermatologists to reduce possible differences, and standardize the results by alleviating inter- and intra-observer variations, and accelerate the process [7].

At the first stage of dermoscopy image analysis, border detection is usually applied [8]. Since the human eye does not perceive minor color and shape changes, there are many factors that make automated border detection complex. For instance, low contrast between the surrounding skin and the lesion, fuzzy and irregular lesion border, and intrinsic artifacts such as cutaneous features (air bubbles, blood vessels, hairs and black frames) are some of the complex cases [7]. According to Celebi et al. [16] automated border detection can be divided into four sections: pre-processing, segmentation, post-processing, and evaluation. The pre-processing step involves color space transformation [9], contrast enhancement [10] and artifacts removal [11]. The segmentation step involves partitioning of an image into disjoint regions [12]. The post-processing is used to obtain the lesion border [8]. The evaluation involves the evaluation of the border detection results made by the dermatologist.

The DBSCAN clustering algorithm, introduced in 1996 [14], is generally used for discovering clusters in large spatial databases with noise. A recent approach for lesion border detection in dermoscopy images, fast density-based lesion detection [13], obtained one of the most accurate results. In that study [13], a modified version of prominent density based clustering algorithm, DBSCAN with the pre-processing step. This approach is a fast density based lesion detection (FDBLD) which removes redundant computations in DBSCAN by selectively picking querying points, core points (see section FDBLD for algorithmic details).

In this paper, the focus is on FDBLD to further improve accuracy of the algorithm for detection of lesion border in dermoscopy images. FDBLD is highly depended on pre-processing step. In the pre-processing step the intermeans algorithm is used to create a binary image [17]. Since a binary image and Manhattan distance are used in FDBLD, one of the most important components of ABCDE mnemonic, C (color) is missed. Moreover, previous approach is heavily dependent on the results of the pre-processing step. If another segmentation technique is used for pre-processing, even for the same image FDBLD tends to generate different results. Therefore, the primary focus on this study is two-fold: first, removing dependency of FDBLD in the pre-processing step; thus, using color information, and second, improving accuracy of the results. To achieve this, new distance measure is incorporated in to FDBLD.

In the following section DBSCAN and FDBLD algorithms are introduced. Next, normalized distance is introduced, and updated FDBLD is introduced. Finally, the experiments and results section compares normalize distance embedded FDBLD (ND-FDBLD) against FDBLD.

## Density-based clustering: DBSCAN

DBSCAN [14] is a notable clustering algorithm. It requires two parameters namely epsilon (ε) and a  minimum number of points (MinPts). DBSCAN is based on a key idea: to form a new cluster or grow an existing cluster the ε-neighborhood of a point should contain at least a minimum number of points, *MinPts*. Neighbors of a point P are those points that are close to the point P. The neighborhood of a point is determined by choice of a distance function regarding two points in search space, such as Euclidean. Searching for ε-neighborhood requires a region query, which is, in 2D, to look for neighboring points in ε-radius around a query point. The major advantage of DBSCAN is that it can follow the arbitrary shapes of the clusters and requires only a distance function and two input parameters: ε and *MinPts*. A detailed theoretical formulation is given in [14].

**Figure 1:**
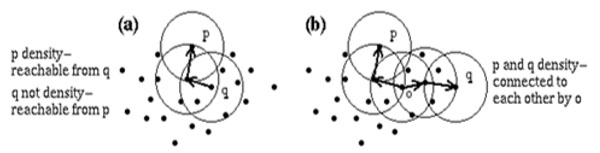
Density-reachability and density-connectivity

Once the two parameters ε and *MinPts* are set, DBSCAN starts to cluster data points from an arbitrarily chosen point *P*. It begins with finding the neighbors of point *P in* ε-neighborhood, i.e., all points that are directly density reachable from point *P* (see Figure 1). If the neighborhood is sparsely populated, i.e., it has fewer neighbors than *MinPts*, point *P* is labeled as noise. Otherwise, a new cluster is initiated and all points in ε-neighborhood of point *P* are marked by the new cluster's label. Next, the neighborhoods of all *P’*s neighbors are examined iteratively to check if new candidates can be added into the cluster. If a cluster cannot be expanded further, DBSCAN chooses another arbitrary unlabeled point (if any such point exists) and repeats the same procedure to form another cluster. These search-and-create procedures are iterated until all data points in the dataset have been labeled as noise or with a cluster label. The major drawback of DBSCAN is that for a dataset containing *n* points, *n* region queries are required to fire during cluster creation.

Regarding a thresholded (binary) image *I*, let its dimension be N × N. For a pixel *p*, let *p_x_* and *p_y_* denote its position where top-left corner is (0, 0) of *I*. Let *c_xy_* = {0,1} represent the value of pixel *p* at (*p_x_*,*p_y_*). Also, let foreground be zero-valued pixels, *c_xy_* = 0. The ε-neighborhood of a pixel *p*, denoted by *NEps*(*p*), is defined by

where *dist*() is Euclidean distance, which gives distance between pixels p and q, and given as

By having *NEps*(*p*) one can create a cluster if |*NEps*(*p*)| ≥ *MinPts*. This final check guarantees that the ε-neighborhood of *p* is dense enough to form and expand a cluster. As mentioned above, this process continues until all pixels are queried. Finally, noise in dataset (pixels without any cluster label) is assumed not to be part of the foreground. The following summarizes DBSCAN algorithm:

**a) ***Eps:* Maximum radius of the neighborhood to be considered while forming clusters.

**b) ***MinPts:* Minimum number of points required to form a cluster.

**c) ***Eps-neighborhood*: A point q is said to be in the Eps-neighborhood of the point p, if the distance between p and q is less than or equal to Eps.

**d) ***Core points and Border points*: Points inside the cluster are called core points and points on the border of the cluster are called border points.

**e) ***Directly density-reachable*: A point q is directly density-reachable from a point p w.r.t ε and MinPts, if q belongs to the Eps-neighborhood of p and the number of points in the ε -neighborhood of p is greater than or equal to MinPts (see Figure 1). If p and q are core points, then directly density-reachable is symmetric i.e., p is directly density-reachable from q and vice versa. However, this condition fails if either p or q is a border point.

**f) ***Density-reachable*: A point p is density-reachable from a point q w.r.t ε and MinPts, if there exists a set of points between q and p such that every point in this set is directly density-reachable from the preceding one.

**g) ***Density-connected*: If there exists a point x such that the points, p and q are both density-reachable from x, then p is said to be density-connected to q w.r.t ε and MinPts (see Figure 1).

**h) ***Noise:* Noise is a set of points in a database that does not belong to any cluster. These points are also called *outliers*.

**i) ***Distance Function:* Distance function is used for determining how much closer two objects are from each other by using all attributes.

This clustering algorithm follows the procedure of finding all points density-reachable from an arbitrary starting point, depending on the ε and MinPts. If the starting point is a core point then the procedure begins building a cluster. Core point is a point which has more than MinPts points around its ε-neighborhood. On the other hand, if the processed point is a border point the algorithm cannot go further, i.e., DBSCAN cannot find any point density-reachable from the starting point. This procedure is followed until all of the points in the Eps-neighborhood are touched or visited at least once. After all of the points in a cluster are visited, the algorithm chooses a new arbitrary starting point to generate other clusters.

## Boundary based clustering: FDBLD

DBSCAN spends most of its computational time in region queries. As mentioned in the previous section, the major drawback of DBSCAN for a dataset containing *n* points, *n* region queries are required to complete clustering. Therefore, any improvement in decreasing the number of neighborhood searches would be beneficial in terms of the efficiency of the algorithm. To this end, FDBLD targets the problem of the excessive number of region queries fired in clustering process. For instance, a very large number of region queries becomes more problematic in the case of applications like virtual slides [18].

Although FDBLD can be generalized for higher dimensional datasets, the applications in 2D are a primary focus of this study. The idea of FDBLD in 2D is to rely on the cluster's boundary, which is a novel concept. Having these boundaries, we can identify those points that are likely to change current shape, the border of the cluster. In DBSCAN as well as in FDBLD, the area of a cluster always expands out and never shrinks. In the case of queries that cannot affect the cluster's area, looking for the ε-neighborhood is treated as unnecessary and omitted in FDBLD. This improvement certainly is very advantageous for the running time of the algorithm since unnecessary computations are removed. The idea behind determining the border of a cluster is derived from the border of a primitive cluster.

***Definition 1*:** Primitive cluster, *PC,* is a cluster formed by a core point and bounded by a convex hull.

**Figure 2:**
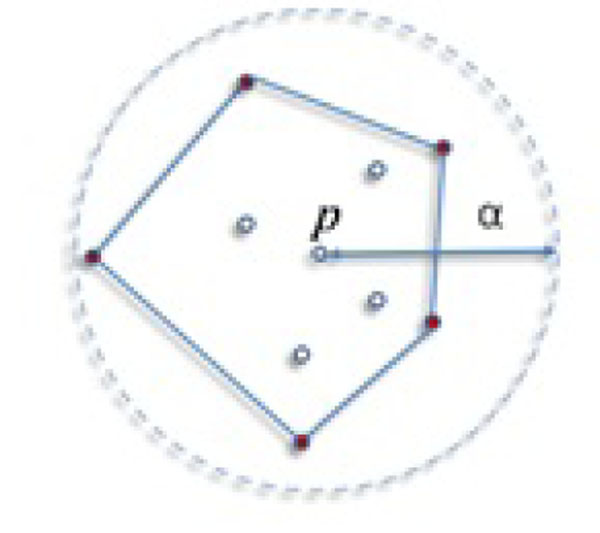
Convex hull which represents a primitive cluster. MinPts = 5.

As seen in [14], each cluster formation starts with a core point, which is also the method to create core point candidates. To keep the boundary of a cluster, we represent ε -neighborhood of each core point with a convex hull, which is a special simple polygon. The convex hull encloses all points found in the neighborhood including query point *p*. Figure 2 shows a primitive cluster around the center point *p*, which is a core point for the given problem. Once the query is fired around *p*, we find 9 points (excluding the query point itself) which are more than *MinPts* of this sample. The convex hull serves as a boundary of *PC*. Note that the schema seen in Figure 2 can be observed at any time during the cluster expansion.

### Expanding cluster in FDBLD

Clustering involves the expansion of the first PC. Once the first core point in the dataset forms a convex hull, it becomes initial boundary of a cluster. Afterward, each of the convex hulls of core points is combined with main body of the cluster. Principally, this operation corresponds to the union of two polygons.

**Figure 3:**
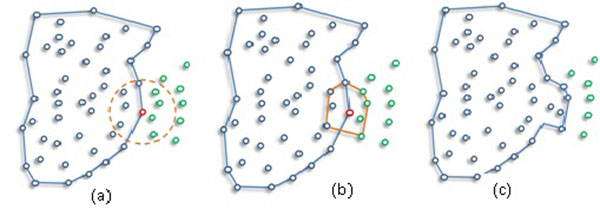
Expanding a cluster

Adding a convex area can expand the cluster in various ways. Figure 3 shows how a newly found convex hull joins the main body of a cluster in three steps. The ε -neighborhood query (dashed line) in Figure 3 (a) a query (red) point satisfies the *MinPts* condition; thus, four new points will be added into the existing cluster. Edges of the primitive cluster around the red point, the convex hull in Figure 3 (b), change the boundary of the current cluster by merging with the current cluster. The final appearance of the cluster's boundary is indicated in Figure 3 (c). The expansion of cluster iteratively continues by examining other points in the region of leading points until no more unlabeled point is found. The points that are not associated with any cluster are labeled as noise, as it is in DBSCAN.

**Figure 4:**
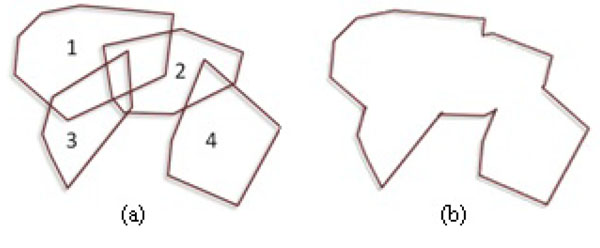
Unionized convex hulls generate a polygon.

A simple polygon is the first step in the cluster formation and does not consider donut-like clusters. *PCs* iteratively form polygon Γ around data points. In Figure 4 (a), assume that *PC*1 is the first *PC* formed in the dataset. Since at this time there is no Γ to be merged, *PC*1 becomes Γ at the same time. Once *PC*2 is obtained, it is unionized with Γ to expand it. After two more iterations for *PC*3 and *PC*4, the final Γ is given in Figure 4 (b). Although one polygon is enough for the boundary of a simple cluster(SC), more simple polygons are needed to represent donut-like clusters.

### Selecting leading points

FDBLD differs from DBSCAN in selecting points in order to expand a cluster. Throughout clustering, DBSCAN fires ε-neighborhood query for each point *P* in seed list of a growing cluster regardless of its impact on current contours of the cluster. It means that ε-neighborhood queries of the points that cannot alter the boundary of a cluster would waste computational time. Obviously, some of the queries would make changes to the shape while others that are relatively far (ε far or ε-width) from the edges would not. On the other hand, it is important to note that most of the expansions made by a query are not final, and these changes will not be seen in the latest structure of the cluster.

FDBLD only fires queries that potentially change the boundaries of a cluster rather than firing queries for each in the dataset. To select the leading points, the outlined algorithm keeps the boundaries of the polygons that delineate the cluster body. Speed ups gained with FDBLD compared to DBSCAN is given in our previous study. Interested readers are referred to [13] and [22]. Contrary to DBSCAN, we do not inspect the status of the points, whether they are core or border points, where the label of these points does not have any contributions in terms of the result of clustering. In FDBLD, the cluster body can enlarge only through points that are qualified for ε-neighborhood queries. Hence, if a point is close enough to a cluster boundary, we fire a ε-neighborhood search around it, otherwise no query will be fired for it. Note; however, it does not mean that every query will alter the shape of a cluster. Therefore, we maintain the set of points that are likely to change the boundaries of a cluster.

**Figure 5:**
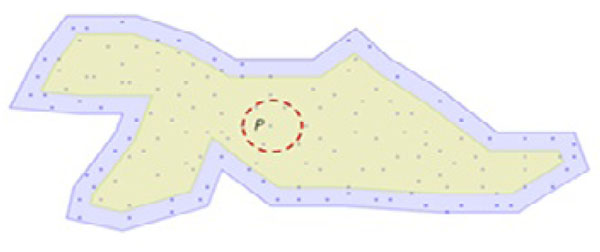
Leading points (blue region).

As seen in the simple cluster in Figure 5, the leading data points are only found in the border region (blue region in Figure 5) including the points on the edges of the outer polygon. The points in the yellow area cannot modify the boundary of growing cluster due to the ε-neighborhood. For instance, point P and its ε-neighborhood dashed circle of Figure 5 cannot alter the boundary of the cluster; thus, this query will be skipped. Actually, all of the queries for the points in the yellow region in Figure 5 will be skipped since they cannot alter the cluster. For this reason, the region of leading points, which includes all leading points, can be imagined as an ε-width inner band (blue region in Figure 5) around the Γ of the cluster C.

### FDBLD algorithm

**Figure 6:**
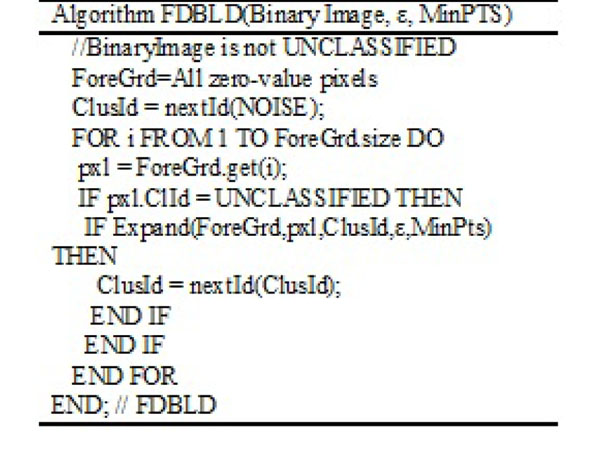
Algorithm FDBLD

**Figure 7:**
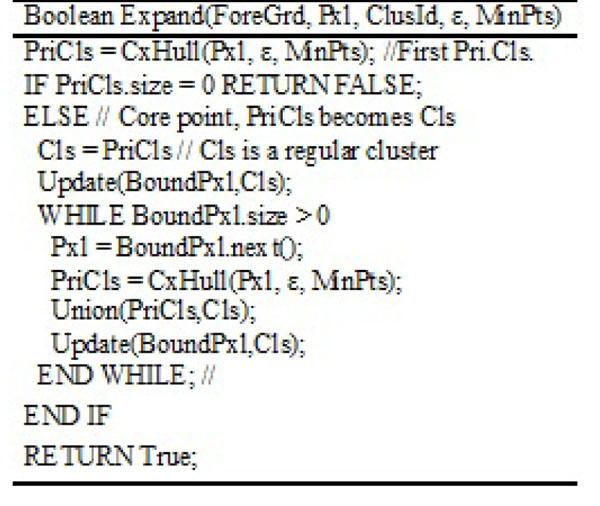
Function expand for FDBLD

The algorithm of FDBLD in 2D is given in Figure 6. Output is the number of clusters found in the image. The major function of FDBLD is Expand which is given in Figure 7. The boundary of each cluster is obtained from Cls variable, which includes at least one simple polygon.

By firing ε-neighborhood query around Pxl, CxHull calculates the boundary of Primitive Cluster, PriCls. If the first PriCls is a null pointer structure, Expand returns false for this query pixel. Otherwise, the cluster Cls is formed from PriCls. The next step is to expand as long as the list of boundary pixels is not empty. Union reshapes the current Cls by unionizing PriCls and Cls. Finally, update functions take a list of boundary pixels and current cluster Cls, and return updated boundary pixels. Usually many of pixels are removed from the list because of expansion of the Cls.

## Normalized distance

In this section, a modified normalized distance measure in Euclidean space, which also includes spatial properties of points (pixels in this case), is proposed. The following is a proposed modified normalized distance measure for RGB color space and pixel coordinates:

where d(i,j) represents normalized Euclidean distance between pixels i and j. In the equation, w_3_, w_4_, and w_5_ are weights for individual R, G, and B channels respectively, where initially w_3_=w_4_=w_5_=1. We introduce different weights for different color channels (for future uses) since in some application domains certain color channels have more significant impacts than others. However, w_1_ is weight for RGB color channels against (x,y) coordinates and w_2_ is weight for (x,y) coordinates for pixels against RGB color channels, where 1/w_1_ + 1/w_2_ = 1, by default w_1_ = w_2_. For instance, in some cases if spatial position (x,y coordinate) is more important than color information, then w_1_ should be greater than w_2_. In that case, the difference in pixel positions will have a greater impact on distance calculation. ΔR, ΔG, ΔB, Δx, Δy in order represents RGB channel differences and x, y spatial coordinate differences between two pixels. 3 x 255^2^ is RGB channel normalization constant. ω is width and h is height of image for normalization of spatial distance (Δx^2^+ Δy^2^).

If we generalize the modified normalized distance measure for multispectral images, it becomes as following:

where k is the multispectral channel number, n is the total number of channels, ΔC_k_ represents channel k the difference between i and j, w_k_ represents weights for each channel where . In this way, both color and spatial position information are included in a single distance metric. With this distance measure, it becomes possible to differentiate even the same colored pixels in different locations.

**Figure 8:**
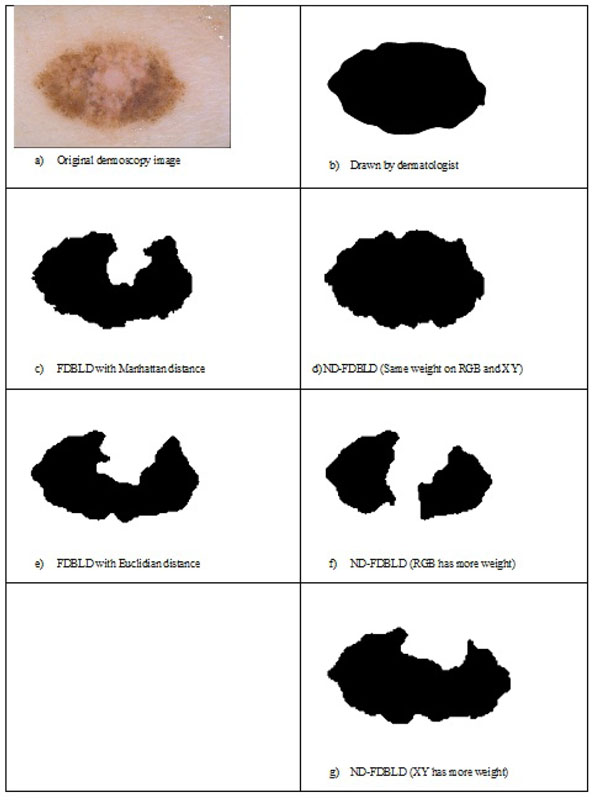
Results generated by FDBLD with different distance measures

Figure 8 illustrates the importance of normalized distance measure over results. Figure 8 a) shows an exemplary dermoscopy image of a lesion which has low contrast between the surrounding skin and the lesion and also a fuzzy and irregular lesion border. Figure 8 b) illustrates the result drawn by a dermatologist whereas Figure 8 c) illustrates the result generated by FDBLD with Manhattan distance while Figure 8 d) illustrates the result generated by ND-FDBLD. As clearly seen from this example, the simple normalized distance measure was specifically designed for lesion border detections (ND-FDBLD) outperforms FDBLD. Figure 8 e) shows the result of FDBLD with Euclidean distance (after preprocessing is applied). Figure 8 f) and g) illustrate how ND-FDBLD behaves with different weights.

Moreover, the normalized distance (ND) makes a finding common ε value for all images very simple. ε interval is found in a few trials. With FDBLD without pre-processing, it is not possible to find common ε interval which generates satisfactory results for all images. With ND, FDBLD works directly on color images without any pre-processing (intermeans segmentation) step.

## Experiments and results

Initially, we studied on determining the two main parameters, MinPts and Eps, of clustering algorithm. An empirical process was used to determine these two parameters. We randomly selected 16 dermoscopy images in order to find the correct values for MinPts and Epsilon. After the parameters are determined, new ND embedded FDBLD is tested on 100 dermoscopy images. Table 1 illustrates the difference between ND embedded FDBLD and FDBLD in Mete et al. [11]. The first column of the table is image ID’s of 100 dermoscopy images. Each object in an image is labeled after applying the clustering algorithm. The results indicate two labels, cancer and non-cancer. For this comparison, precision, recall, and border error are used. We calculated these measurements by using the formula below.

**Table 1:** Comparison between ND-FDBLD and FDBLD with respect to error rate, precision, and recall.

Img.ID	ND-FDBLD			FDBLD			Img.ID	ND-FDBLD			FDBLD		
	**B. Err.**	**Pre.**	**Rec.**	**B. Err.**	**Pre.**	**Rec.**		**B. Err.**	**Pre.**	**Rec.**	**B. Err.**	**Pre.**	**Rec.**

**1**	0.05	0.96	0.99	0.03	1.00	0.88	**51**	0.09	1.00	0.91	0.03	1.00	0.93
**2**	0.08	0.92	0.98	0.02	0.94	0.86	**52**	0.08	1.00	0.92	0.05	1.00	0.83
**3**	0.08	0.96	0.96	0.09	0.89	0.76	**53**	0.04	1.00	0.96	0.02	0.99	0.90
**4**	0.04	0.97	1.00	0.08	0.98	0.79	**54**	0.04	0.97	1.00	0.09	1.00	0.73
**5**	0.06	0.95	0.99	0.04	1.00	0.76	**55**	0.05	0.96	1.00	0.08	1.00	0.75
**6**	0.04	0.98	0.98	0.05	0.98	0.86	**56**	0.03	0.99	0.97	0.05	1.00	0.81
**7**	0.06	0.95	0.99	0.08	0.93	0.87	**57**	0.05	1.00	0.95	0.06	1.00	0.83
**8**	0.04	0.96	1.00	0.05	0.89	0.85	**58**	0.03	1.00	0.97	0.05	1.00	0.83
**9**	0.04	0.97	0.98	0.06	1.00	0.84	**59**	0.05	1.00	0.95	0.01	1.00	0.96
**10**	0.06	0.94	1.00	0.06	1.00	0.86	**60**	0.05	0.98	0.97	0.03	1.00	0.91
**11**	0.10	0.91	1.00	0.04	1.00	0.84	**61**	0.06	0.95	1.00	0.14	1.00	0.62
**12**	0.03	0.98	1.00	0.04	0.96	0.89	**62**	0.03	0.99	0.98	0.07	1.00	0.81
**13**	0.04	0.96	1.00	0.03	1.00	0.88	**63**	0.02	0.98	0.99	0.06	1.00	0.81
**14**	0.08	0.93	1.00	0.03	1.00	0.85	**64**	0.03	1.00	0.97	0.03	1.00	0.81
**15**	0.02	0.98	0.99	0.02	1.00	0.93	**65**	0.01	1.00	0.99	0.01	1.00	0.92
**16**	0.01	1.00	0.99	0.01	0.99	0.94	**66**	0.02	0.99	1.00	0.05	0.90	0.80
**17**	0.06	0.94	1.00	0.08	1.00	0.57	**67**	0.03	0.97	1.00	0.05	1.00	0.77
**18**	0.06	0.96	0.98	0.11	1.00	0.68	**68**	0.02	0.98	1.00	0.04	1.00	0.81
**19**	0.13	0.89	1.00	0.13	1.00	0.72	**69**	0.01	1.00	0.99	0.01	1.00	0.90
**20**	0.02	1.00	0.98	0.05	1.00	0.71	**70**	0.03	1.00	0.97	0.02	1.00	0.80
**21**	0.03	0.99	0.98	0.05	1.00	0.80	**71**	0.03	0.98	0.99	0.06	1.00	0.68
**22**	0.01	0.99	0.99	0.04	1.00	0.76	**72**	0.04	0.96	1.00	0.10	1.00	0.68
**23**	0.02	0.99	0.99	0.04	1.00	0.85	**73**	0.05	0.99	0.96	0.05	0.94	0.77
**24**	0.02	0.98	1.00	0.06	1.00	0.71	**74**	0.01	0.99	1.00	0.02	0.99	0.85
**25**	0.03	1.00	0.97	0.05	1.00	0.87	**75**	0.07	0.94	1.00	0.08	1.00	0.65
**26**	0.04	1.00	0.97	0.05	1.00	0.85	**76**	0.40	0.72	1.00	0.11	1.00	0.71
**27**	0.04	0.97	0.98	0.07	1.00	0.82	**77**	0.01	0.99	1.00	0.03	1.00	0.73
**28**	0.03	0.99	0.99	0.06	1.00	0.82	**78**	0.11	0.90	1.00	0.13	1.00	0.62
**29**	0.05	0.96	0.99	0.07	1.00	0.76	**79**	0.14	0.88	1.00	0.12	1.00	0.69
**30**	0.02	0.98	1.00	0.05	1.00	0.80	**80**	0.04	0.96	1.00	0.07	1.00	0.63
**31**	0.33	0.75	1.00	0.33	1.00	0.52	**81**	0.01	1.00	1.00	0.02	1.00	0.61
**32**	0.04	0.96	1.00	0.08	1.00	0.76	**82**	0.14	0.88	1.00	0.12	1.00	0.74
**33**	0.06	0.94	1.00	0.06	1.00	0.70	**83**	0.05	0.96	1.00	0.11	1.00	0.52
**34**	0.04	0.97	0.99	0.08	1.00	0.79	**84**	0.01	0.99	1.00	0.03	1.00	0.78
**35**	0.05	0.98	0.97	0.06	1.00	0.83	**85**	0.04	0.97	0.99	0.08	1.00	0.76
**36**	0.11	0.90	1.00	0.07	1.00	0.77	**86**	0.05	0.98	0.97	0.09	0.98	0.76
**37**	0.03	0.98	1.00	0.09	1.00	0.80	**87**	0.03	0.98	0.99	0.07	1.00	0.73
**38**	0.04	0.96	1.00	0.02	0.99	0.90	**88**	0.02	0.98	1.00	0.06	1.00	0.55
**39**	0.03	0.98	0.99	0.03	1.00	0.90	**89**	0.03	0.99	0.98	0.04	0.89	0.90
**40**	0.01	1.00	0.99	0.02	1.00	0.92	**90**	0.07	0.95	0.98	0.17	1.00	0.55
**41**	0.03	0.99	0.97	0.05	1.00	0.82	**91**	0.03	0.97	1.00	0.08	1.00	0.61
**42**	0.02	1.00	0.98	0.03	1.00	0.88	**92**	0.06	0.97	0.98	0.05	1.00	0.88
**43**	0.02	1.00	0.98	0.06	1.00	0.76	**93**	0.02	0.98	1.00	0.02	1.00	0.90
**44**	0.04	1.00	0.96	0.02	1.00	0.86	**94**	0.06	0.96	0.98	0.15	1.00	0.65
**45**	0.01	0.99	1.00	0.04	1.00	0.82	**95**	0.01	1.00	0.99	0.03	1.00	0.66
**46**	0.04	0.96	1.00	0.08	1.00	0.73	**96**	0.06	0.98	0.96	0.09	1.00	0.74
**47**	0.02	1.00	0.98	0.03	1.00	0.85	**97**	0.31	0.76	1.00	0.23	1.00	0.65
**48**	0.04	0.97	1.00	0.08	1.00	0.73	**98**	0.04	0.96	0.99	0.05	1.00	0.83
**49**	0.05	0.96	1.00	0.15	1.00	0.73	**99**	0.05	0.95	1.00	0.12	1.00	0.64
**50**	0.01	0.99	1.00	0.04	1.00	0.83	**100**	0.03	0.97	1.00	0.03	1.00	0.70

Precision=tp/(tp+fp) , Recall= tp/(tp+fn)

Border error (BE) measure, which is also called XOR measure, was first developed by Gao et al. [17]. This measure quantifies the percentage of border detection error. It is the most commonly used measure and accepted by the skin lesion detection researchers. Thus, XOR measure is more important for skin lesion detection researchers than precision and recall. It was first used by Hance et al. [19]. Schaefer et al. [20][21] also uses XOR measure for dermoscopy images, and it is calculated by:

**BE**=[**(AB**⊕**MB)*/*MB**]**×100**

where ⊕ is exclusive OR operator, essentially underlines disagreement between the target (ManualBorder, MB) and predicted (AutomaticBorder, AB) regions. Referring to information retrieval terminology, the nominator of the BE means summation of false positive (FP) and false negative (FN). The denominator is obtained by adding true positive (TP) to false negatives (FN). After the pixels in an image are labeled, the number of true prediction of the lesion area was named true positive, the number of false prediction of lesion area as false positive, the number of true prediction of non-lesion as true negative, and the number of false prediction of non-lesion as false negative. Better results were obtained than FDBLD in 75 images among 100 images. In Table 1, ND embedded FDBLD is compared against the original FDBLD on the same dataset. The three columns following the first column show our results and the second three columns represent results from Mete at al.[11].

**Figure 9:**
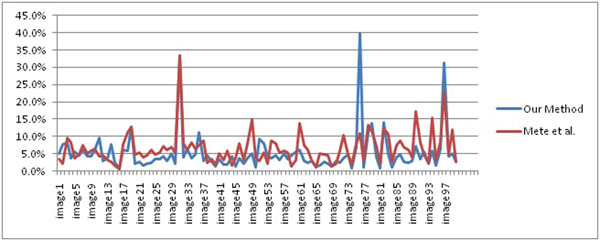
Comparison between our method and FDBLD (Mete et al. [13]), x direction is image numbers, y direction is border error of corresponding image.

As seen from Table 1, the proposed lesion border detection method is more accurate than FDBLD. However, as seen from Figure 9, FDBLD outperforms our method in two images which are image numbers 76 and 97. In these images, there exists a cutaneous feature which is hair. In these images, hair occludes the lesion area and they elongate all the way down to the image borders which are considered as peripherals in the authors' approach. Therefore, hairs which intersect the lesion area and the regions residing between the hairs are included in the lesion area. These cutaneous features will be taken in to consideration in our future studies.

## Conclusions

A new distance measure, so-called normalized distance, is embedded in the previous study which used a novel density based clustering algorithm for automated lesion border detection in dermoscopy images. This measure not only considers pixel positions but also considers pixel colors for distance computation. A recent study of the fast density based lesion border detection (FDBLD) approach by Mete et al.[13], was used as a basis for this study. Accuracy of the FDBLD was greatly dependent on the preprocessing step. To improve accuracy of the FDBLD and remove its dependency to the preprocessing step, a new distance measure is integrated to the existing approach.

Results show that dependency of FDBLD to the pre-processing step is discarded by integrating normalized distance measure to it. Moreover, efficiency of the FDBLD is improved by achieving lower border error rates for automated lesion border detections in dermoscopy images. The proposed method was tested on 100 dermoscopy images. Results were compared with both FDBLD and manually drawn lesion borders by dermatologists for the same images. In order to measure the accuracy of the obtained results, precision, recall, and border error rate measures were used. The results show that normalized distance measure embedded FDBLD better performs in 75% of dermoscopy images than the original study and this new approach reduces overall border error rates.

## Competing interests

The authors declare that they have no competing interests in regards to this study.

## Authors' contributions

SS and SK have made equal contributions to this study. SS has made implementations. SS and SK have made analysis. SK designed normalized distance measure. MM provided data and previously published study's results in order to make comparisons.

## References

[B1] National Cancer Institute Common Cancer Types http://www.cancer.gov/cancertopics/commoncancers Accessed June 2011

[B2] Jemal A Siegel R Xu J Ward E 2010 Cancer Statistics CA Cancer J Clin 2010 277 300 DOI: 10.3322/caac.20073 10.3322/caac.20073 20610543

[B3] American Cancer Society Cancer Facts&Figures 2010 http://www.cancer.org/acs/groups/content/@nho/documents/document/acspc-024113.pdf Accessed June 2011

[B4] Rigel DS Carucci JA Malignant melanoma: prevention, early detection, and treatment in the 21st century CA Cancer J Clin 2000 5 no:4, 50 215 236 10.3322/canjclin.50.4.215 10986965

[B5] Lorentzen H Weismann K Petersen CS Larsen FG Secher L Skødt V Clinical and dermatoscopic diagnosis of malignant melanoma. Assessed by expert and non-expert groups Acta dermato venereologica 1999 79 4 3014 doi:10.1080/000155599750010715. PMID 10429989 10.1080/000155599750010715 10429989

[B6] Binder M Schwarz M Winkler A Steiner A Kaider A Wolff K Pehamberger H Epiluminescence microscopy. A useful tool for the diagnosis of pigmented skin lesions for formally trained dermatologists Arch. Dermatol 1995 131 3 286 291 10.1001/archderm.131.3.286 7887657

[B7] Celebi ME Iyatomi H Schaefer G Stoecker WV Lesion border detection in dermoscopy images Comput. Med.Imag. Graphics 2008 33 148 153 10.1016/j.compmedimag.2008.11.002 PMC2671195 19121917

[B8] Celebi ME Aslandogan YA Bergstresser PR Unsupervised border detection in dermoscopy images Skin Res. Technol 2005 2 123 128

[B9] Pratt WK Digital Image Processing: PIKS Inside 2007 John Wiley and Sons (Jun 2006), Hoboken, NJ, USA doi: 10.1002/9780470097434.ch5

[B10] Gomez DD Butakoff C Ersboll BK Stoecker W Independent histogram pursuit for segmentation of skin lesions IEEE Trans. Biomed. Eng 2008 55 157 161 18232357 10.1109/TBME.2007.910651 PMC3161407

[B11] Celebi ME Kingravi HA Iyatomi H Aslandogan YA Stoecker WV Moss RH Malters JM Grichnik JM Marghoob AA Rabinovitz HS Menzies SW Border detection in dermoscopy images using statistical region merging Skin Res. Technol 2008 14 347 353 10.1111/j.1600-0846.2008.00301.x 19159382 PMC3160669

[B12] Sonka M Hlavac V Boyle R Image processing, analysis, and machine vision Cengage-Engineering 2007 third

[B13] Mete M Kockara S Aydin K Computerized Medical Imaging and Graphics 2010 35 2 128 136 10.1016/j.compmedimag.2010.07.007 20800995

[B14] Ester M Kriegel HP Sander J Xu X A density-based algorithm for discovering clusters in large spatial databases with noise Conference on Knowledge Discovery and Data Mining 1996 DOI: 10.1.1.71.1980

[B15] Lim JS Two-Dimensional Signal and Image Processing 1990 Englewood Cliffs, NJ, Prentice Hall 469 476

[B16] Celebi ME Lesion border detection in dermoscopy images Comput. Med. Imag. Graphics 2009 33 148 153 10.1016/j.compmedimag.2008.11.002 PMC2671195 19121917

[B17] Otsu N A threshold selection method from gray-level histogram IEEE Trans. Systems Man Cybernet 1979 9 62 66

[B18] Sertel O Computer-aided prognosis of neuroblastoma on whole-slide images: Classification of stromal development Pattern Recognition 2009 42 no. 6 1093 1103 10.1016/j.patcog.2008.08.027 20161324 PMC2678741

[B19] Hance GA Umbaugh SE Moss RH Stoecker WV Unsupervised Color Image Segmentation with Application to Skin Tumor Borders IEEE Engineering in Medicine and Biology 1996 15 1 104 111 10.1109/51.482850

[B20] Schaefer G Skin lesion extraction in dermoscopic images based on colour enhancement and iterative segmentation Proceedings of IEEE International Conference on Image Processing (ICIP 2009). Cairo, Egypt 2009 3361 3364

[B21] Schaefer G Skin lesion segmentation using cooperative neural network edge detection and colour normalisation Proceedings of the Ninth International Conference on Information Technology and Applications in Biomedicine (ITAB 2009). Larnaca, Cyprus 2009

[B22] Kockara S Mete M Chen B Aydin K Analysis of density based and fuzzy c-means clustering methods on lesion border extraction in dermoscopy images BMC Bioinformatics 2010 11 S26 20946610 10.1186/1471-2105-11-S6-S26 PMC3026373

